# The Association between Serum 25-Hydroxyvitamin D Concentrations and Depressive Symptoms in Korean Adults: Findings from the Fifth Korea National Health and Nutrition Examination Survey 2010

**DOI:** 10.1371/journal.pone.0099185

**Published:** 2014-06-19

**Authors:** Hye-Kyung Chung, Yoonsu Cho, Sumi Choi, Min-Jeong Shin

**Affiliations:** 1 Severance institute for vascular and metabolic research, College of Medicine, Yonsei University, Seoul, Republic of Korea; 2 Department of Food and Nutrition, Korea University, Seoul, Republic of Korea; 3 Department of Public Health Sciences, Graduate School, Korea University, Seoul, Republic of Korea; 4 Department of Child Development and Family Studies, Pusan National University, Pusan, Republic of Korea; 5 Korea University Guro Hospital, Korea University, Seoul, Republic of Korea; University of Medicine & Dentistry of NJ - New Jersey Medical School, United States of America

## Abstract

The aims of this study were to examine the association between circulating vitamin D (25(OH)D) levels and depressive symptoms and to evaluate the associations between depressive symptoms and various sociodemographic factors. Data on serum 25(OH)D levels, sociodemographic factors, and information on depressive symptoms were obtained from the Korea National Health and Nutrition Examination Survey V-1 2010. A total of 3,570 Koreans aged ≥20 years were included in the statistical analysis. Subjects with depressive symptoms had lower serum levels of 25(OH)D (41.6±16.2 nmol/L) than those without (44.3±16.2 nmol/L; P-value<0.05; effect size = 0.17). In a logistic regression analysis, the 25(OH)D sufficiency group (≥50 nmol/L) revealed fewer depressive symptoms (OR, 0.72; 95% CI, 0.53–0.97; P-value = 0.032) after adjusting for multiple factors. In addition, females (OR, 3.61; 95% CI, 2.55–5.11; P-value<0.001), problematic alcohol users (OR, 2.33; 95% CI, 1.63–3.34; P-value<0.001), current smokers (OR, 1.43; 95% CI, 1.02–1.99; P-value = 0.036), and subjects who experienced weight loss (OR, 1.78; 95% CI, 1.30–2.44; P-value<0.001) were more likely to answer “yes” on question for depressive symptoms. In conclusion, low serum levels of 25(OH)D were associated with an increased risk for depression symptoms in Korean adults. In addition, several sociodemographic factors were related to the depressive symptoms. Our results provide insight into the relationships among vitamin D status, sociodemographic factors, and depression in the Korean population.

## Introduction

Depression is an important health problem that threatens health and quality of life worldwide. The World Health Organization has reported that depression is a major contributor to disability [1]. The reports of the Global Burden of Disease pointed out the rising burden from mental and behavioral disorders, which have increased in all years of life living with a disability composition of 38% from 1990 to 2010 [2]. Thus, the socioeconomic loss due to depression is substantial and similar to the burden of cancer and cardiovascular disease [3], [4]. In Korea, 7.9% of males and 16.9% of female adults aged >19 years have experienced depressive symptoms according to Korea Health Statistics 2010 [5]. In addition, the suicide rate in Korea is number one in the Organization for Economic Cooperation and Development states [6]. Given that depression is one of the strongest risk factors for suicide [7], healthcare plans to prevent and reduce depression are particularly needed for Koreans.

It is well known that 25-hydroxyvitamin D (25(OH)D) is involved in calcium homeostasis and maintaining bone health [8], [9]. In addition, accumulating data indicate that levels of 25(OH)D are associated with many diseases such as cancer, diabetes, hypertension, coronary vascular disease, obesity, and asthma [10]–[15]. Emerging experimental evidence suggests that metabolites of 25(OH)D can cross the blood brain barrier and 25(OH)D receptors exist in the central nervous system [16], [17], which raises the possibility that 25(OH)D might be involved in brain and cognitive function [17], [18]. Several studies have inconsistently reported that lower levels of serum 25(OH)D are significantly associated with depression [11], [19]–[22]. In contrast, recent studies have failed to show a relationship between 25(OH)D levels and depression in elderly subjects [23] or healthy women [24]. This discrepancy among study results could be, in part, due to the small numbers of subjects or that sociodemographic factors were not comprehensively considered when determining the associations.

In the present study, we evaluated whether circulating 25(OH)D levels are associated with depressive symptom after adjusting for multiple confounding factors using large-scale data from the 5th Korea National Health and Nutrition Examination Survey (KNHANES V-1). In addition, we evaluated the associations between depressive symptoms and various sociodemographic factors such as age, gender, body mass index, marital status, education, income levels, experience with body weight control, perceived body shape, alcohol behavior, smoking status, and physical activity in this population.

## Subjects and Methods

### Study Population

**Figure 1:**
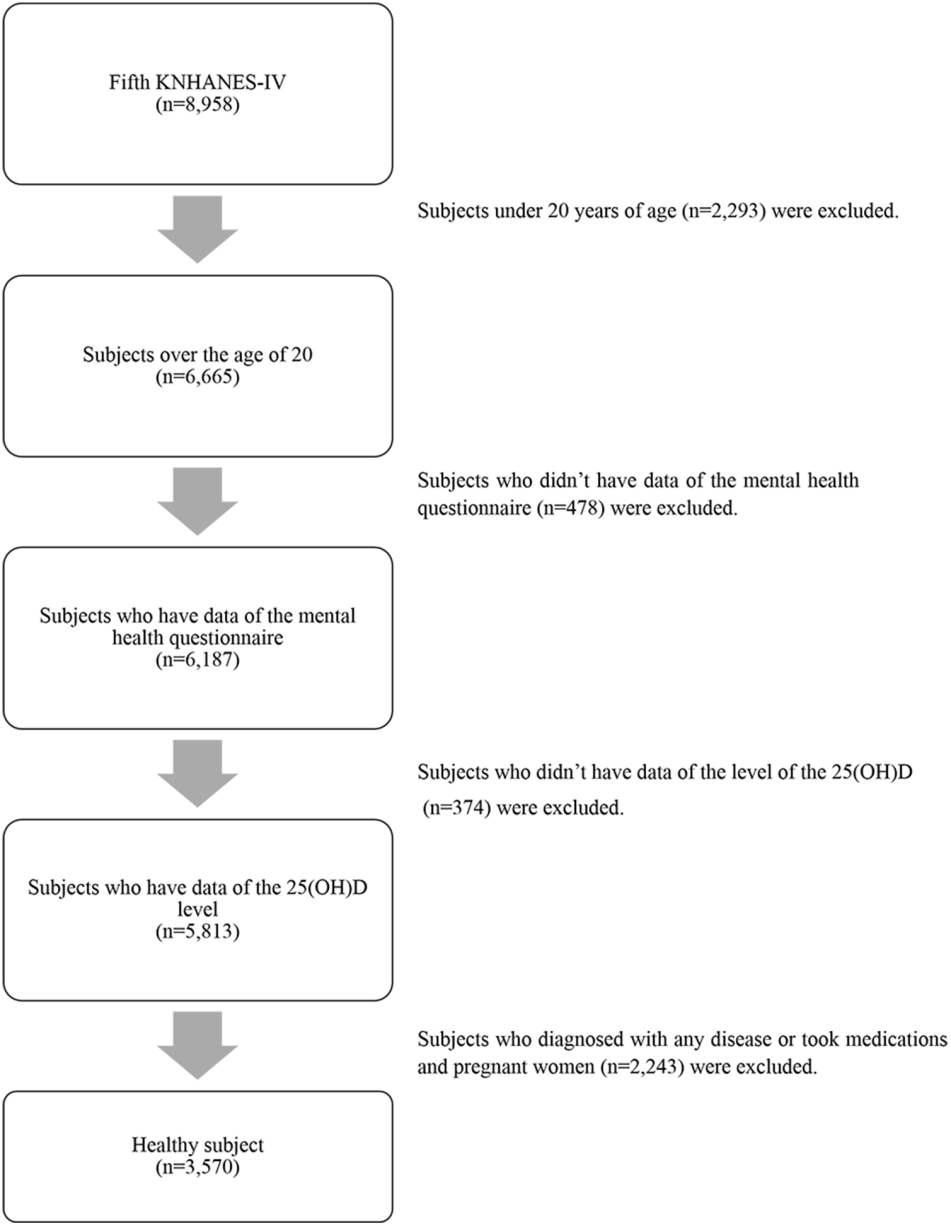
Flow chart of study population.

This study was based on data from the KNHANES V-1, 2010. The KNHANES is a cross-sectional survey conducted nationwide by the Division of Korean National Health and Welfare. The KNHANES is composed of three different sections: a health interview, a health examination, and a nutrition survey. The 5th KNHANES (2010) was performed from January 2010 to December 2010. The survey includes a representative national sample of the Korean population, choosing from recorded households in the Population and Housing Census in Korea in 2005. The survey section is arranged by district and housing type characteristics. Twenty households were selected from each survey section using a stratified, multistage probability cluster sampling method that considers geographical area, age, and gender. In the 5th KNHANES (2010), 10,938 individuals aged ≥1 year were sampled for the survey. Among them, 8,958 individuals participated in the examination (response rate of 81.9% for aged ≥1 year). Of the 8,958 subjects who participated in the KNHANES, we limited the analyses to adults aged ≥20 years (Fig. 1). We excluded subjects whose data were missing for important analytic variables, such as serum 25(OH)D levels and the mental health questionnaire. Additionally, we excluded subjects with chronic diseases, including stroke, hypertension, type 2 diabetes mellitus, ischemic heart failure, cirrhosis, arthritis, asthma, renal failure, cancers, and thyroid disease to eliminate factors affecting 25(OH)D metabolism or depression. Pregnant or lactating female subjects were also excluded because of unique changes in hormones. Finally, 3,570 subjects were included in the statistical analysis. The KNHANES was approved by the Institutional Review Board of the Centers for Disease Control and Prevention in Korea. All participants in the survey signed an informed written consent form.

### General Characteristics of the Subjects

We obtained data from KNHANES V-1 including demographic, anthropometric, nutritional, and personal medical history data. Anthropometric measurements were obtained by trained experts following standardized protocols. Body weight and height of subjects were measured to the nearest 0.1 kg and 0.1 cm, respectively. Body mass index (BMI) was calculated as weight (kg)/height squared (m^2^). BMI was classified as underweight (<18.5), normal weight (18.5≤25) or obese (≥25). Demographic variables that were expected to be confounding included gender, age, marital status, education, monthly income, experience with body weight control, perceived body shape, alcohol behavior, smoking status, physical activity and examination date. Marital status was divided into two groups of single or married based on questionnaire responses. Education level was divided into four groups: elementary school, middle school, high school, or university. Monthly income was divided into quartiles and reported in the South Korean currency won as follows: lowest (≤1 million won), lower middle (1 million won ≤2 million won), upper middle (2 million won ≤3 million won), or highest (>3 million won). Body weight control meant that subjects had experienced trying to gain or lose weight and was divided into four groups: weight-loss, maintaining, weight-gain, or none. Perceived body shape was divided into lean, normal, or obese. We used data from the Alcohol Use Disorders Identification Test (AUDIT) to assess alcohol use behaviors of subjects [25]. Referring to a previous study [26], subjects were classified into three groups according to their AUDIT score using tertile values: normal user (0–7), hazardous user (8–14), or problematic user (15–40). Current smokers were defined as a person who smoked cigarettes at present. Physical exercise was divided into two groups such as: practice or do not practice, according to whether or not the individual participated in any of the following activity types for 5 days per week; intense physical activity at least 20 minutes, moderate physical activity at least 30 minutes, or walking at least 30 minutes. Examination date was categorized into four groups depending on the season: Spring (March–May), summer (June–August), fall (September–November), or winter (December–February).

### Psychological Health

The psychological health data were obtained from a self-reported mental health questionnaire under the supervision of an investigator and was a section of the health interview. Depressive symptoms included feeling sad or despair continuously for >2 weeks and enough to interfere with daily life during the last year. Subjects were divided into 2 groups according to their answer, “depression” or “non-depression”. The validity of the assessment method for depressive symptoms was evaluated by previous studies [27], [28].

### Serum 25(OH)D Assessment

Blood samples were collected through an antecubital vein after 10–12 h of fasting to assess serum levels of biochemical markers. Serum levels of 25(OH)D were measured by gamma counter with a radioimmunoassay (25-hydroxy-vitamin D125 I RIA Kit; DiaSorin, Stillwater, MN, USA) using a 1470 Wizard Gamma Counter (PerkinElmer, Turku, Finland). To minimize the analytical variation, serum 25(OH)D levels were analyzed by the same institute, which carried out a quality assurance program through the analysis period. The interassay coefficients of variation were 1.9–6.1% for the samples. The subjects were subdivided into two groups according to general guideline [29] for serum 25(OH)D in determining the association between serum 25(OH)D levels and depressive symptoms: 25(OH)D insufficiency (<50 nmol/L), or 25(OH)D sufficiency (≥50 nmol/L).

### Statistical Analysis

All statistical analyses were performed using SPSS ver. 21.0 (SPSS, Inc., Chicago, IL, USA). Continuous variables are described as means ± standard deviations and categorical variables are expressed as numbers and percentages of subjects. Each variable was tested for normality before statistical analysis. Logistic regression analysis was conducted to evaluate associations between depressive symptom and serum 25(OH)D concentration or sociodemographic factors. Odds ratios (ORs) for depressive symptoms were compared between 25(OH)D insufficiency group and 25(OH)D sufficiency group. Two models were constructed for logistic regression. One was analyzed without adjustment and the other was adjusted for gender, age, BMI, education, body weight control, perceived body shape, AUDIT score, smoking status, physical activity, and season. Confounding factors that could affect both serum levels of 25(OH)D and depressive symptom were defined by previous findings. Previous studies reported that serum levels of 25(OH)D are associated with age, sex, BMI, marital status, education, income, experience with body weight control, perceived body shape, alcohol behavior, smoking status, activity levels and seasonal changes [30]–[39]. Thus, all of these variables were considered as confounding factors to analyze the association between serum levels of 25(OH)D and depressive symptom. Subjects with missing values for any covariates were excluded in the multivariate logistic regression analysis. Explorative P-values<0.05 were considered significant.

## Results

### General Characteristics of the Subjects

The mean age of all subjects was 43.2±13.8 years (46.9% males and 53.1% females). The general characteristics of the study subjects are shown in Table 1. Approximately 68% of all subjects had a normal weight, and 80.8% were married. University graduates accounted for 40.2%, followed by high school graduates at 38.6%, elementary school graduates at 12.7%, and middle school graduates at 8.6%. Approximately 30% of the subjects were in the highest income group and 12.0% were in the lowest income group. Normal alcohol users accounted for 63.4%, followed by hazardous users at 19.1%, and problematic users at 17.5%. Current smokers and subjects with depressive symptom included 26.1% and 10.8% of all subjects, respectively. 49.3% of subjects exercised at least five days per week.

**Table 1:** General characteristics of the study subjects.

		Total (n = 3570)
BMI	Under weight	186 (5.2)^1^
	Normal weight	2414 (67.8)
	Obese	962 (27.0)
Marital status	Single	684 (19.2)
	Married	2885 (80.8)
Education	Elementary school	450 (12.7)
	Middle school	304 (8.6)
	High school	1369 (38.6)
	University	1427 (40.2)
Income	Lowest	425 (12.0)
	Lower middle	929 (26.3)
	Upper middle	1112 (31.5)
	Highest	1062 (30.1)
Body weight control	Weight-loss	1453 (40.7)
	Maintaining	584 (16.4)
	Weight-gain	208 (5.8)
	Not at all	1320 (37.0)
Perceived body shape	Lean	626 (17.6)
	Normal	1433 (40.2)
	Obese	1506 (42.2)
Alcohol behavior	Normal user	2036 (63.4)
	Hazardous user	612 (19.1)
	Problematic user	561 (17.5)
Smoking status	Non-smoker	2634 (73.9)
	Current smoker	930 (26.1)
Physical activity	No	1809 (50.7)
	Yes	1761 (49.3)
Depressive symptoms	No	3179 (89.2)
	Yes	384 (10.8)

1 Values are number of subjects in the total of study population; n (%). Alcohol behavior, alcohol use disorders identification test (AUDIT) to assess the alcohol use behaviors of subjects; Smoking status, smoking cigarettes at present; Physical activity, practice severe physical activity at least 20 minutes, moderate physical activity at least 30 minutes, or walk at least 30 minutes, 5 days per week; Depressive symptoms, feeling sad or despair continuously for >2 weeks and enough to interfere with daily life during the last year.

### Association between Serum 25(OH)D Levels and Depressive Symptoms

We evaluated the association between serum 25(OH)D levels and depressive symptoms. Subjects with depressive symptoms had lower serum levels of 25(OH)D (41.6±16.2 nmol/L) than those without these psychological problems (44.3±16.2 nmol/L; P-value<0.05; effect size = 0.17). Table 2 shows the logistic regression analysis of the association between serum 25(OH)D levels and depressive symptoms. The ORs for depressive symptoms were calculated according to the serum 25(OH)D level: 25(OH)D insufficiency (<50 nmol/L), or 25(OH)D sufficiency (≥50 nmol/L). The 25(OH)D sufficiency group was associated with fewer depressive symptoms before (OR, 0.69; 95% CI, 0.54–0.88; P-value = 0.003) and after adjustment (OR, 0.72; 95% CI, 0.53–0.97; P-value = 0.032).

**Table 2:** Odds ratios and 95% confidence intervals for the depression symptoms according to serum 25(OH)D cutoff value level.

	Serum 25(OH)D (nmol/L)		P for trend
	<50.0	≥50.0	
Depressive symptom			
Unadjusted^1^	1.00	0.69 [0.54–0.88]	0.003
Multivariate-adjusted^2^	1.00	0.72 [0.53–0.97]	0.032

1 Differences were tested using unadjusted logistic regression analysis.

2 Differences were tested using multivariate-adjusted logistic regression analysis after adjusting for gender, age, BMI, income, education, marital status, body weight control, perceived body shape, alcohol behavior, smoking status, physical activity, and season.

### Associations between Socioeconomic Factors and Psychological Health

We further tried to evaluate the relationships between various socioeconomic factors and depressive symptoms (Table 3). In a logistic regression analysis after adjusting for covariates, females (OR, 3.61; 95% CI, 2.55–5.11; P-value<0.001), problematic alcohol users (OR, 2.33; 95% CI, 1.63–3.34; P-value<0.001), current smoker (OR, 1.43; 95% CI, 1.02–1.99; P-value = 0.036), and subjects who experienced weight loss (OR, 1.78; 95% CI, 1.30–2.44; P-value<0.001) are more likely to answer “yes” on question for depressive symptoms. The Subject who did physical activities (OR, 0.74; 95% CI, 0.59–0.95; P-value = 0.016) and subject with high levels of education showed lower rate for positive answer to the question for depressive symptoms (OR, 0.49; 95% CI, 0.30–0.80; P-value = 0.005).

**Table 3:** Odds ratios and 95% confidence intervals for perceived stress and depression symptoms according to sociodemographic factors.

	Depressive symptom	P-value^3^
Age^1^	1.00 [0.99–1.02]	0.675
Gender^2^		
Male	-	-
Female	3.61 [2.55–5.11]	<0.001
BMI^1^	0.99 [0.94–1.04]	0.649
Marital status^2^		
Single	-	-
Married	0.81 [0.56–1.16]	0.248
Income^1^	0.89 [0.79–1.01]	0.077
Education^2^		
Elementary school	-	-
Middle school	0.75 [0.46–1.24]	0.268
High school	0.58 [0.37–0.92]	0.021
University	0.49 [0.30–0.80]	0.005
Body weight control^2^		
Not at all	-	-
Weight-loss	1.78 [1.30–2.44]	<0.001
Maintaining	1.03 [0.67–1.53]	0.868
Weight-gain	1.18 [0.66–2.08]	0.579
Perceived body shape^1^	0.84 [0.66–1.08]	0.185
Alcohol behavior^2^		
Normal user	-	-
Hazardous user	1.23 [0.87–1.74]	0.240
Problematic user	2.33 [1.63–3.34]	<0.001
Smoking status^2^		
Non-smoker	-	-
Current smoker	1.43 [1.02–1.99]	0.036
Physical activity^2^		
No	-	-
Yes	0.74 [0.59–0.95]	0.016

1 Differences were tested using continous variable.

2 Differences were tested using categorial variable.

3 Differences were tested using logistic regression analysis. Differences were tested using multivariate logistic regression analysis after adjusting for gender, age, BMI, income, education, marital status, body weight control, perceived body shape, alcohol behavior, smoking status, physical activity, and season.

## Discussion

In the present study, we investigated whether circulating levels of 25(OH)D are associated with depressive symptoms in Korean healthy population. Consistent with several earlier findings [21], [30], [40]–[43], the results showed that serum 25(OH)D levels were associated with depressive symptoms. A prospective study of overweight and obese adults demonstrated that subjects with low 25(OH)D levels have a higher degree of depression [30]. A recent observational study on the elderly and a large study in US adults demonstrated the relationship between 25(OH)D deficiency and depression [40], [41]. In another study, adolescents with psychotic features reveal lower serum levels of 25(OH)D [43]. A small-scale cross-sectional studies showed that subjects with schizophrenia or major depression had lower levels of 25(OH)D_3_ and 1,25(OH)_2_ D_3_ [21], [42]. Study conducted in patients with mild Alzheimer’s disease and non-demented subjects reported that those with low 25(OH)D (<20.0 ng/mL) were more likely to have a mood disorder [19]. Furthermore, premenopausal women with major depressive disorder (MDD) show lower 25(OH)D levels compared to those of controls [21]. In a large observational study, patients with MDD or minor depression showed lower mean 25(OH)D levels than those of a control [22]. Jorde et al. demonstrated that 25(OH)D levels are negatively correlated with the presence of depression as assessed by the Beck Depression Inventory (BDI) score [20]. However, conflicting data have also been published [23], [44]. While the exact mechanism of the association between vitamin D and mental health remains elusive, our results provide additional evidence supporting that circulating 25(OH)D concentration is associated with depressive symptoms in healthy adults, but are distinct from an earlier finding in that the associations were evident even after adjusting for multiple confounding factors in a large-scale Korean population.

Of interest is the observed association between sociodemographic factors and depressive symptoms. Logistic regression analysis revealed that females, problematic alcohol users, and smokers had more depressive symptoms. Women have a higher tendency to display an emotional coping style than men, and as a result, women may experience psychological distress more often than men [45]. A 21-year longitudinal study suggested that major depression was associated with increased rate of daily smoking [46], which is consistent with our finding. On the other hand, physical activity may help to reduce depressive symptoms. Previous study with MDD patient showed that depressive symptoms and anxiety state were significantly reduced after exercise intervention [47]. More interestingly, subjects who tried to lose weight had higher levels of depressive symptoms. As seen in the result of Jung’s research about the relationship of age and life satisfaction [48], such a low level of physical satisfaction results in low self-esteem and life satisfaction. Also, there is evidence that low educational levels were significantly associated with both anxiety and depression due to their failure in social and economic activities [49], implicating that general life satisfaction is related to depressive symptom. It can be suggested that the link between sociodemographic factors and depressive symptoms might be associated with the relations of socioeconomic factors to circulating 25(OH)D which also shows strong correlation with depressive symptoms. Indeed, we also observed that serum levels of 25(OH)D were associated with multiple sociodemographic factors. Serum levels of 25(OH)D were associated with age, body mass index, marital status, education, income, experience with body weight control, perceived body shape, alcohol behavior, smoking status, and physical activity (all P-values<0.05, Table S1). Considering the interrelationships among sociodemographic factors, circulating 25(OH)D and psychological health indicators, our results suggest that various socio-demographic factors could be associated with serum 25(OH)D as well as depressive symptoms, and that these factors seem to coexist.

Serum 25(OH)D levels depend on both sunlight and dietary intake. In the present study, serum 25(OH)D was higher in subjects who conducted regular physical activity but was not associated with intake frequency of 25(OH)D rich food (data not shown). These results may reflect that 25(OH)D biosynthesis by sunlight exposure plays a primary role compared to intake of 25(OH)D source food in this population. Previous studies have supported that lack of sunlight exposure is the main cause of vitamin D deficiency because very few foods have high vitamin D content [50]–[52]. It can also be speculated that subjects with depression are reluctant to engage in outdoor activity [22], [31], which can lead to decreased chance for sunlight exposure, resulting in lower serum 25(OH)D levels. In particular, average serum 25(OH)D levels (44.0 nmol/L) were lower in Koreans than those in non-Hispanic whites [41]. In line with this statements, 69.8% of our subjects had lower levels of 25(OH)D, under 50 nmol/L, which is the desirable minimal level of serum 25(OH)D to minimize the risk for osteomalacia and rickets [52]–[53]. Therefore, increasing vitamin D synthesis through outside activity is recommended to maintain appropriate levels of 25(OH)D considering the association between levels of 25(OH)D and depressive symptoms in Koreans.

Our study had several limitations. First, this was a cross-sectional designed study; thus, we could not clarify cause-and-effect relationships between serum 25(OH)D levels and mental health. Second, depressive symptoms were assessed simply by self-reported questionnaires: “Have you felt sad or desperate continuously for ≥2 weeks so as to disturb your everyday life?” Accuracy of a single question in screening for depression reported through the previous studies [27], [28], yet, this concern still remains a controversy. Third, the amount of dietary vitamin D intake was not present in the KNHANES V-1 data. Additionally, this exclusion of the subjects might limit the generalizability of our findings to different population. Finally, the subjects with minor gastrointestinal disorders have not been dealt with confounding factor who have possibility of low serum levels of 25(OH)D due to poor absorption of dietary Vitamin D [54]. Despite these limitations, our study has important meaning in that it is the first study to find that serum 25(OH)D is associated with depressive symptoms after adjusting for multiple potential confounding factors in Koreans.

In conclusion, low serum levels of 25(OH)D were associated with an increased risk for depression in Korean adults. In addition, depressive symptoms were associated with sociodemographic factors, including gender, alcohol behavior, smoking, exercise, and experience with weight loss. Our results provide insight into the relationships among vitamin D status, sociodemographic factors and depression.

## Supporting Information

Table S1 **Serum 25(OH)D levels according to demographic characteristics.** Serum levels of 25(OH)D were associated with age, body mass index, marital status, education, income, experience with body weight control, perceived body shape, alcohol behavior, smoking status, and physical activity. Values are expressed as mean ± standard deviation. Differences were tested by ANOVA with Bonferroni’s multiple comparisons test or t-test. Different letters indicates significant differences between the two groups.
(DOCX)
