# Supplementary material for: The Association between Serum 25-Hydroxyvitamin D Concentrations and Depressive Symptoms in Korean Adults: Findings from the Fifth Korea National Health and Nutrition Examination Survey 2010
Source: PLoS One. 2014 Jun 19;9(6):e99185. doi: 10.1371/journal.pone.0099185 (PMC4063710; doi:10.1371/journal.pone.0099185)
Supplement: Table S1 — Serum 25(OH)D levels according to demographic characteristics. Serum levels of 25(OH)D were associated with age, body mass index, marital status, education, income, experience with body weight control, perceived body shape, alcohol behavior, smoking status, and physical activity. Values are expressed as mean ± standard deviation. Differences were tested by ANOVA with Bonferroni’s multiple comparisons test or t-test. Different letters indicates significant differences between the two groups. (DOCX) [file pone.0099185.s001.docx]

Table S1. Serum 25(OH)D levels according to demographic characteristics.

|  | | Serum 25(OH)D (nmol/L) | |
| --- | --- | --- | --- |
|  | | Total (n=3570) | P-value |
| BMI | Under weight (n=186) | 41.0±18.3^a^ | 0.024^4^ |
|  | Normal weight (n=2414) | 44.1±16.4 ^b^ |  |
|  | Obese (n=962) | 44.5±15.2 ^b^ |  |
| Marital status | Single (n=684) | 39.3±14.4 | <0.001^5^ |
|  | Married (n=2885) | 45.2±16.4 |  |
| Education | Elementary school (n=450) | 51.0±20.2 ^a^ | <0.001^4^ |
|  | Middle school (n=304) | 47.9±17.6 ^a^ |  |
|  | High school (n=1369) | 42.9±15.4 ^b^ |  |
|  | University (n=1427) | 42.2±14.5 ^b^ |  |
| Income | Lowest(n=425) | 47.7±18.8 ^a^ | <0.001^4^ |
|  | Lower middle(n=929) | 44.5±16.0 ^b^ |  |
|  | Upper middle (n=1112) | 42.5±15.3 ^c^ |  |
|  | Highest(n=1427) | 43.9±16.0 ^bc^ |  |
| Body weight control | Weight-loss (n=1453) | 42.8±15.0 ^a^ | 0.003^4^ |
|  | Maintaining (n=584) | 44.9±16.5 ^ab^ |  |
|  | Weight-gain (n=208) | 45.2±17.2 ^ab^ |  |
|  | Not at all (n=1320) | 44.9±17.1 ^b^ |  |
| Perceived body shape | Lean (n=626) | 45.8±17.7 ^a^ | <0.001^4^ |
|  | Normal (n=1433) | 44.6±17.1 ^a^ |  |
|  | Obese (n=1506) | 42.7±14.5 ^b^ |  |
| Alcohol behavior^1^ | Normal user (n=2036) | 42.7±15.7 ^a^ | <0.001^4^ |
|  | Hazardous user (n=612) | 44.9±15.6 ^b^ |  |
|  | Problematic user (n=561) | 47.9±16.9 ^c^ |  |
| Smoking status^2^ | Non-smoker (n=2634) | 43.5±16.0 | 0.001^5^ |
|  | Current smoker (n=930) | 45.6±16.7 |  |
| Physical activity^3^ | No (n=1809) | 42.9±15.9 | <0.001^5^ |
|  | Yes (n=1761) | 45.2±16.4 |  |
| Season | Spring (n=857) | 39.2±13.2 ^a^ | <0.001^5^ |
|  | Summer (n=973) | 46.5±16.9 ^b^ |  |
|  | Fall (n=840) | 51.0±17.0 ^c^ |  |
|  | Winter (n=900) | 39.5±14.3 ^ad^ |  |

Values are expressed as mean ± standard deviation

^1^ Alcohol behavior: alcohol use disorders identification test (AUDIT) was used to assess the alcohol use behaviors of subjects. ^2^ Smoking status: smoking cigarettes at present. ^3^ Physical activity: practice severe physical activity at least 20 minutes, moderate physical activity at least 30 minutes, or walk at least 30 minutes, 5 days per week. ^4^ Differences were tested by ANOVA with Bonferroni’s multiple comparisons test. Different letters indicates significant differences between the two groups.^5^ Differences were tested by Student’s*t*-test (P-value <0.05).
